# Cell and biomaterial-based approaches to uterus regeneration

**DOI:** 10.1093/rb/rbz021

**Published:** 2019-04-26

**Authors:** Feiran Liu, Shiqi Hu, Shaowei Wang, Ke Cheng

**Affiliations:** 1 Department of Gynecology and Obstetrics, Beijing Hospital, National Center of Gerontology, Beijing, China; 2 Department of Molecular Biomedical Sciences and Comparative Medicine Institute, North Carolina State University, Raleigh, NC, USA; 3 Joint Department of Biomedical Engineering, University of North Carolina at Chapel Hill and North Carolina State University, Raleigh, NC, USA; 4 Division of Pharmacoengineering and Molecular Pharmaceutics, Eshelman School of Pharmacy, University of North Carolina at Chapel Hill, Chapel Hill, NC, USA

**Keywords:** Asherman’s syndrome, intrauterine adhesions, endometrium, stem cells

## Abstract

Asherman’s syndrome (AS) is an endometrial disorder in which intrauterine adhesions crowd the uterine cavity and wall. The fibrotic adhesions are primarily the result of invasive uterine procedures that usually involve the insertion of surgical equipment into the uterus. This syndrome is accompanied by a number of clinical manifestations, including irregular or painful menstruation and infertility. The most prevalent treatment is hysteroscopy, which involves the physical removal of the fibrous strands. Within the last decade, however, the field has been exploring the use of cell-based therapeutics, in conjunction with biomaterials, to treat AS. This review is a recapitulation of the literature focused on cellular therapies for treating AS.

## Introduction

Stem cell therapies have been well documented in the scientific literature as promising and versatile alternatives to traditional, pharmaceutical paradigms. To date, numerous maladies have been modeled in animals to test the efficacy and safety of a range of cellular therapies. These include myocardial infarction [1], neurodegenerative disorders [2], osteoarthritis [3], liver fibrosis [4] and lung injury [5], among others. Many of these therapies have even gone on to be tested in humans in a number of clinical trials [6]. A more recent disease model, Asherman’s syndrome (AS), has emerged in the field of obstetrics and gynecology (ObGyn) as a possible candidate for stem cell-driven regenerative treatment.

AS, also referred to as intrauterine adhesions (IUAs), or intrauterine synechiae, is a complex gynecological abnormality caused by procedures that impart damage to the endometrium, such as dilation and curettage (D&C), hysteroscopic resection, hysteroscopic septum resection, hysteroscopic myomectomy and abdominal myomectomy [7]. Symptoms include recurrent pregnancy loss, hypomenorrhea, amenorrhea and infertility [8]. There are a number of treatments available to patients with AS, including hormone therapy, hysteroscopic surgery and the insertion of intrauterine devices. However, the causes and severity of the IUAs vary among patients. Thus, the efficacy of these treatments is not always very obvious and can be quite variable from patient to patient. In this review, we will summarize the most up-to-date research available on the application of cellular therapy as a treatment option for AS.

Stem cells exhibit self-renewal and multilineage differentiation potential. Among them, mesenchymal stem cells (MSCs), which are extracted from adipose tissue, bone marrow, the placenta, peripheral blood, endometrium and umbilical cords, have unique immunomodulatory properties and intrinsic heterogeneity [9]. As a result, they have been widely used in tissue regeneration and organ repair with promising and/or successful outcomes. There is increasing evidence supporting the theory that paracrine signaling molecules secreted by the MSCs, which include a broad range of cytokines, chemokines and growth factors, are responsible for repairing the injury.

During the last decade, many scholars have fixed their sights on using MSCs to restore uterine health and reverse the damage caused by AS. A large number of animal experiments and a limited number of clinical trials have reported successful outcomes (Table 1), but there are still many questions left unanswered. In this review, we will shed light on the progress made thus far, the direction this field of research is taking and the lessons we can learn from other stem cell therapies.

**Table 1.** Summary of endometrial cellular studies

Year published	Cell line	Model	References
2011	Human endometrial side population	NOD-SCID mice	10
	Autologous bone marrow cells	Human patient	11
2014	Male wistar albino rat MSCs	Wistar Albino rats	12
	Male C57BL/6 mouse bone marrow-derived stem cells	B57BL/6 mice	13
2015	Human CD133+ bone marrow-derived stem cells	Non-obese diabetic mice (strain code 394; NOD.CB17-Prkdcscid/NcrCrl)	14
2016	CD133+ bone marrow-derived stem cells	Human patient	15
2017	Autologous bone marrow mononuclear cells with collagen scaffold	Human patient	16
2018	Allogeneic umbilical cord MSCs	Human patient	17
	Sprague–Dawley bone marrow MSCs	Sprague–Dawley rats	18
	C57BL/6-Tg mouse bone marrow-derived cells and uterine-derived cells	Transgenic C57BL/6J mice expressing-enhanced GFP	19

## The disease—AS

The uterus is comprised of three tissue layers. The inner-most layer is the endometrium. It consists of the functional and basal endometrium. When the functional layer is shed, it results in menstrual bleeding. If there is damage in the basal layer of the endometrium, adhesions and fibrosis can form. In 1894, Heinrich Fritsch was the first to describe IUAs, also known as synechiae, after noticing secondary amenorrhea in a patient who underwent a postpartum curettage [8]. The ‘AS’ nomenclature was later adopted in honor of Joseph Asherman. In 1948, he described the frequency and etiology of the condition. To differentiate it from other conditions, he specified that, in addition to IUAs, factors such as infertility, cyclic pelvic pain and menstrual disturbance had to be considered [8]. The terms IUAs and AS are still used interchangeably, but it is important to note that the syndrome requires a number of symptoms in addition to IUAs. Hysteroscopic evaluation is regarded as the most reliable method for diagnosing AS. Although other factors, such as infection, inflammation and retention of trophoblastic tissue contribute to the development of IUAs, AS is most frequently caused as postoperative complications after intrauterine surgery, with D&C procedures being the largest contributors [8]. D&C is a common surgical operation and is frequently performed in women who experience a miscarriage. A meta-analysis comparing 10 individual studies in which 912 women were followed-up 12 months after miscarriage showed that about 19.1% developed IUAs [20].

## Pathological mechanisms

The pathophysiological underpinnings of AS have not been fully elucidated and the literature still provides only limited data. Ongoing research has centered around a few key endometrial observations that shed light on what is driving the formation of the adhesions. Women diagnosed with severe AS exhibit ribosome loss, hypoxic cellular modifications, mitochondrial swelling and vascular closure. In addition, the endometrial environment is characterized by a decreased ability to revascularize and produce new blood vessels, hypoxia and inflammation [8].

Patients with dense IUAs have impaired vascularity in both the myometrium and endometrium. In one prospective study, the uterine spiral arteries in patients with AS exhibited a high impedance [20]. Patients that were treated in this same study demonstrated increased vascular endothelial growth factor (VEGF) output and higher micro-vessel densities, indicative of angiogenesis.

Hypoxia, caused by intrauterine operations or inflammation to the endometrium, may also play a vital role in the formation of AS. TGF-β1 and type 1 collagen are all triggered by hypoxia and are relevant to operative adhesions. TGF-β1 plays a significant role in collagen formation and inhibits its degradation. In addition, IUA patients exhibit an increased production of cytokines related to adhesion. These include platelet-derived growth factor, transforming growth factor-beta and fibroblast growth factor [8].

There is also much interest in a more recent etiological possibility: the injury and subsequent aberrant repair of the endometrial stem cell population. More and more research shows that adult stem cells exist in the uterine endometrium [21]. Endometrial stem cells are adult stem cells that make up only a small percentage of the total cellular population and play an important role in maintaining tissue homeostasis. The first study to demonstrate their existence was published in 2004 and showed that a group of endometrial cells were clonogenic [22]. Similar results were found in murine endometrium. By detecting the slow-cycling of cells expressing undifferentiation markers-c-Kit and POU5F1, Cervelló *et al*. [23] found that endometrial stem cells were located in the lower region of the endometrial stromal compartment of mice. They also found that the percentage of endometrial stem cells in murine models was higher than the stem cell populations of other organs, such as the bone marrow, lungs and the pancreas, but similar to that of the skin. This might be due to the high proliferation rates of the endometrium during the estrous cycles. In another study, researchers isolated endometrial stem cells from the endometrium of 26 women undergoing hysterectomies and found that the clonogenicity of stem cells in the endometrium does not vary during the menstrual cycle, meaning that the clonogenicity of these cells is hormone independent [24].

There are a number of theories regarding the molecular mechanisms involved in AS. NF-κB mRNA and inflammatory factors are found to be elevated in IUA tissues both in AS patients and rats [25]. Zhao *et al*. [16] have demonstrated that ΔNp63 upregulation in the endometrial epithelium significantly reduced the rate of cellular replication. They demonstrated that ΔNp63 upregulation causes an interruption in endometrial growth by rendering cell cycle less responsive to estradiol (E2). After finding that bone marrow stem cells can downregulate the expression of ΔNp63, this group placed a collagen scaffold carrying autologous bone marrow stem cells, onto the inner uterine surface of five AS patients. All the patients had previously undergone procedures to separate their adhesions. After the scaffolds were implanted, they experienced an increased number of glands, cell density and thickness of the endometrium, and all five patients in the group became pregnant spontaneously or by embryo transfer.

## Current treatment options

There are various therapeutic approaches available for repairing the endometrium after injury, or protecting it from recurrent IUAs. The most common among them is hysteroscopic adhesiolysis, but there is also increased interest in anti-adhesives, hormone therapy and intrauterine devices [7, 8]. Additionally, there are a number of exploratory new therapies that are currently being tested on animals and in small scale clinical trials.

### Hysteroscopic adhesiolysis

Hysteroscopic adhesiolysis is a common method used to treat IUAs and reconstruct the uterine cavity. It involves the use of a hysteroscope to view the uterus, and surgical, blunt-end scissors to lyse the fibrotic adhesions. Postoperative outcomes vary depending on the severity of the adhesive disease. Many clinical cases have reported that even after adhesiolysis, women with severe IUAs experience adhesion reformation in as many as 60% of cases, and subsequent miscarriages in as many as 75% [20]. Valle and Sciarra [26] reported that, among patients with severe AS, only 57% can get pregnant even after the uterine cavity was completely restored. The live birth rate among severe AS patients following hysteroscopic adhesiolysis is only 31% [20]. Unfortunately, even women who eventually become pregnant and successfully give live births are susceptible to complications after a hysteroscopy. These include abnormal placentation, preterm delivery, intrauterine growth restrictions and the need for higher rates of cesarean sections.

### Hyaluronic acid gel

Hyaluronic acid gel shows promising efficacy as an anti-adhesive after ObGyn operations. In some rabbit models of AS, total fetal delivery was achieved after using the hyaluronic acid gel postoperatively [27]. Some clinical studies also report that the intrauterine application of cross-linked hyaluronic acid gel after hysteroscopic adhesiolysis can reduce the development of adhesions postoperatively compared with D&C alone [28]. According to the American Fertility Society’s (AFS), and the European Society of Gynecological Endoscopy’s classification of IUAs, the application of hyaluronic acid gel is likely to be associated with lower average adhesion scores. Whether or not the intrauterine application of hyaluronic acid gel can prevent the formation of IUAs is not known, but some clinical studies have confirmed that it can reduce the intensity of IUAs. Researchers are seeking to enhance the therapeutic potential of hyaluronic acid gel by infusing it with drugs that might prevent IUAs, essentially creating a carrier for the sustained release of pharmaceuticals or beneficial cellular by-products.

### Hormone therapy

**Figure 1.**
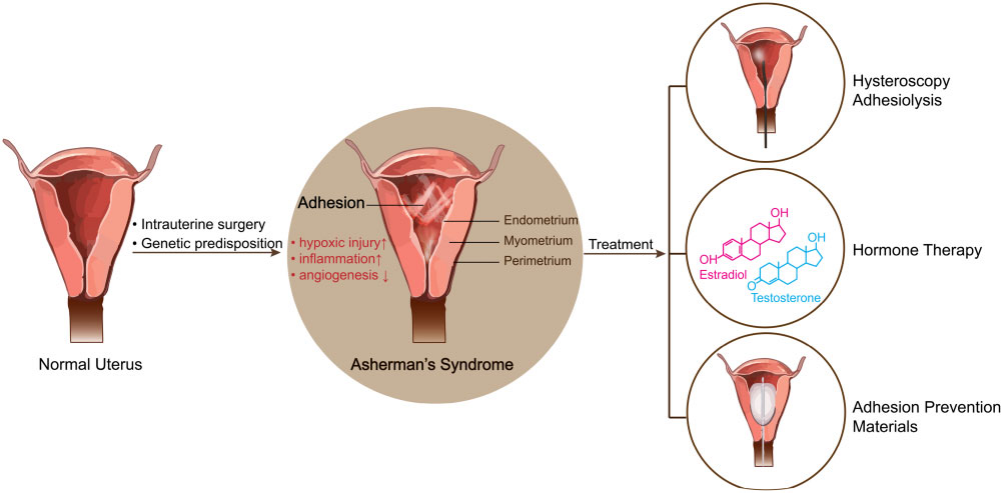
Schematic illustrating pathology and current treatment options for AS. Compared to normal uterine morphology, the pathological changes of AS include formation of IUAs and injury of endometrium, which were induced by hypoxic injury, inflammation and decreased angiogenesis. The current treatment options include hysteroscopy adhesiolysis, hormone therapy and adhesion prevention materials.

17b-Estradiol is an estrogen steroid hormone that is commonly used to treat AS (Fig. 1). It has been shown to effectively improve endometrial thickness and pregnancy rate as well as prevent the recurrence of adhesions after hysteroscopic adhesiolysis. However, it is difficult to find an optimal administration route and dose because of its limited half-life period, poor solubility in aqueous solutions and response differences among patients. Clinically, high doses of estrogen are often applied to patients with IUAs to ameliorate the endometrial injury, but estrogen at high doses may increase the risk of thrombosis and malignancies. The challenge, however, is that low doses of estrogen cannot effectively promote endometrial recovery. Complicating matters further, some studies show no statistical difference between high-dose and low-dose estrogen therapy. Zhang *et al*. [29] encapsulated 17b-estradiol with heparin-poloxamer micelles and formed a thermosensitive, sustained release hydrogel system. The released 17b-estradiol was administered in the uterus to restore the structure and function of the injured endometrium effectively.

### Other treatment options

There are only a few case reports of different treatment options for refractory AS. Aghajanova *et al. *[30] have reported two cases in which the uterine cavity was infused with autologous, platelet-rich plasma after hysteroscopy to improve endometrial growth and function. Platelets in the plasma have been shown to contain alpha-granules, which store cytokines and growth factors involved in cell migration, proliferation, angiogenesis and differentiation, and are released at the site of injury. Thus, it shows a promising applicability in AS. However, the mechanism for how it works is unclear, the number of cases is limited, and no randomized controlled studies have been performed.

Lu *et al*. have reported a prospective, randomized, controlled trial among 80 women who have severe IUA and whose current AFS IUA scores were at least eight [31]. Women in the amnion group were treated with an amnion graft. These were inserted into the uterine cavity for 1 week using a Foley catheter. Three months after surgery, compared to the preoperative value, the amnion group had a lower median AFS score and a substantial improvement in menstruation with respect to women in the control group.

## Regenerative medicine approaches

The field of regenerative medicine, which fuses tissue engineering and molecular biology to repair and regenerate injured tissues or organs, has burgeoned in recent decades. When, in many cases, the only option for terminally ill patients is an organ transplant, regenerative medicine looks for ways in which damaged organs can be restored or induced to self-heal. This has become especially important as the number of patients on organ waiting lists grows, while the supply of said organs remains very limited. In addition, patients who are lucky enough to receive an organ have to consume a host of immunosuppressive drugs for the remainder of their lives to avoid rejection reaction.

Biomaterials, which can mimic the physicochemical properties of cellular or extracellular materials, are regarded as a vital component of regenerative medicine. They can help restore the structure and function of the damaged tissue and can be modified to locally release growth factors or drugs. They can even be loaded with stem cells, another fundamental component of regenerative medicine. Biomaterials can enhance the survival rate of these stem cells and exert a significant influence on their differentiation lineage. A study published in 2006 reported that MSCs respond to biomaterial matrices in both morphology and lineage [32]. Surprisingly, soft matrices, which mimic the brain, proved to induce neurogenic differentiation, while stiffer matrices induced myogenic differentiation. Similarly, rigid matrices, similar to collagenous bone, induced osteogenic differentiation.

Regenerative medicine has many applications in the field of obstetrics as well. Recently, researchers successfully used a ‘cell sheet’ technique to regenerate functional endometrium [33]. The three-layer cell sheet is made of rat endometrial cells. When placed onto the defected area of the endometrium the patch was able to restore the rats’ ability to become pregnant. In another study, researchers constructed a uterine patch from decellularized segments of the uterus. These were then recellularized with MSCs [34]. When transplanted to a defect in the uterus, the patch supported uterine function, including pregnancy. In yet another study, VEGF was bound to collagen to support uterine remodeling [35].

### Biomaterials

The past decade has seen an increase in the number of researchers looking for biocompatible and biodegradable materials that can be used to load drugs to heal IUAs. As mentioned before (pathological mechanisms section), E2-loaded hydrogels are more effective at healing IUAs than E2 delivered alone. Li *et al*. [36] have established a collagen-based basic fibroblast growth factor (bFGF)-targeting delivery system by fusing a collagen-binding domain to bFGF. bFGF is necessary for endometrial remodeling, proliferation and angiogenesis. The collagen-binding domain increases the binding ability of the bFGF to bind to the fibrotic regions of the uterine horn in rats.

### Stem cell therapies

Bone marrow-derived mesenchymal stem cells (BM-MSCs) have the ability to differentiate into many kinds of non-hematopoietic cells, including skeletal myoblasts, cardiac myoblasts and skin epithelia, as well as endothelial, renal, hepatic and lung cells. Their versatility makes them attractive candidates for cell therapies that seek to restore many tissues/organs after injury. More and more scholars are researching the possible therapeutic applications of BM-MSCs for the treatment of AS. Zhang *et al*. [37] have found that MSC-conditioned media promotes human umbilical cord endothelial cell growth and migration, suppresses H_2_O_2_-induced apoptosis of the endometrium and promotes the activation of the AKT and ERK pathways, which are critical for angiogenesis.

Gargett *et al*. [22, 24] has proven that small populations of stromal and epithelial stem cells exist in the uterus. They have found that 1.25% of stromal and 0.22% of epithelial cells exhibit clonogenicity and high proliferative potential. These stem cells are likely to contribute to endometrial proliferation during the menstrual cycle and could decrease during uterine injury. Knowing that there is a native stem cell population in the uterus increases the feasibility of using stem cells to restore uterine injury. Endometrial stem cells reside in the basal layer of the uterus and are thought to be a source of progenitors, with the capacity to differentiate to form the endometrium. Compared to endometrial stem cells, BM-MSCs show greater ability to migrate to the uterus through systemic injections, leading to a higher percentage of donor cells *in situ* [19]. Thus, BM-MSCs have an advantage over endometrial stem cells for undoing uterine damage. When administered by tail vein injection, BM-MSCs not only migrate to the endometrium, but also seem to migrate more successfully in response to injury. It has been demonstrated that under the influence of uterine injury, the engraftment ability of injected BM-MSCs increases by almost 2-fold compared to when they are injected in animals with healthy uteri. Although the injury to the uterus was localized, BM-MSCs migrated to both sides of the uterus, which indicates that the recruitment mechanisms are most likely the result of secreted molecular attractants [38]. The importance of these molecular secretions, specifically paracrine signals, is also a salient research subject for those seeking to understand the mechanisms used by stem cells to impart their therapeutic effects. Interestingly, the fluctuations of systemic hormonal levels had no effect on the migration of BM-MSCs to the uterine endometrium.

### Preclinical studies—IUAs animal model

Establishing an animal model of AS is essential for the study of its pathogenesis and treatments. The methods used to make IUAs can be grouped into three categories: chemical, mechanical and electrical injury. Chemical injury can be achieved through phenol mucilage, trichloroacetic acid and alcohol. However, the most acceptable injury method is mechanical. Feryal *et al.*, for example, used a 27-Gauge needle to cause the uterine injury [13]. They inserted the needle into the uterus through a small incision in the uterine horn, rotating and withdrawing several times, create the uterine synechiae. After three estrous cycles, the disease model was confirmed by histology, using H&E and Masson’s trichrome staining. In addition to chemical and mechanical models, there are a few researchers using electrocoagulation to cause injury. One team used a copper wire, inserted through a small incision in the uterine horn, to deliver 0.5 watts of power over a period of 3 s [37]. Compared to the non-damaged uterine horn, the electro-coagulated endometrium was thinner and had fewer glands, as observed by H&E staining.

### Clinical studies using stem cells

In 2011, a woman received a transvaginal injection of autologous bone marrow stem cells after a curettage left her with severe AS [11]. The curettage had been performed on her during the placement of an intrauterine contraceptive device. Following the stem cell injections, her endometrial thickness increased, vascularity improved, and she was eventually able to maintain embryo growth.

In 2016, researchers published the results of a pilot study that used autologous bone marrow-derived stem cells to treat refractory AS and endometrial atrophy [15]. The novelty of this cohort study was that stem cells were delivered to the endometrial stem cell niche via intra-arterial catheterization. Once the catheter position was verified and fixed, the cells were injected into spiral arterioles. After stem cell transplantation, 15 of the 16 patients enrolled in this trial regained their menstrual cycles. During each of the three hysteroscopic observations conducted after transplantation, all the patients showed an improved endometrium and uterine cavity. Transitory neoangiogenesis was reported to decrease in the third hysteroscopy, 6 months after the operation. Four of the 16 patients became pregnant and delivered a live baby successfully.

It has also been reported that menstrual blood-derived stromal cells (MenSC), which are stem-cell-like cells comprised of a mixture of MSC and stromal fibroblasts derived from menstrual blood, have proven to be effective in the treatment of severe AS [39]. MenSCs were transplanted into the uterus transvaginally, followed by hormonal stimulation. After cell transplantation, the endometria of all the seven patients showed significant proliferation. Five of the seven patients obtained endometrial thicknesses of >7 mm, which is considered adequate for embryo implantation. Three of the seven patients with severe AS became pregnant, with one pregnancy occurring spontaneously.

### The limitations to stem cell therapies

Unfortunately, studies show that stem cells do not just contribute to the proliferation and restoration of normal or injured endometrium. They can also promote ectopic endometrial growth; that is to say, endometriosis. As reported in one of the studies, researchers generated an experimental endometriosis model by implanting wild-type uterine segments to the peritoneal cavity of previously hysterectomized LacZ transgenic mice. LacZ-positive cells with differentiation potential were engrafted in the wild-type ectopic endometrium, and only 0.1% stromal cells and 0.04% epithelial cells were original donor cells in week 10 [40].

Stem cells and cancer cells share a number of characteristics, including a high proliferative capacity, the ability to self-renew and a high degree of plasticity [41]. RNA binding protein, Musashi-1, is considered an immunohistochemical marker specific to endometrial stem cells. Compared to normal secretory endometrium, the cells in endometrial and endometriotic carcinoma tissue show increased Musashi-1 expression, a finding that supports the theory that cancer and endometriosis can occur as a result of aberrant stem cell multiplication. Endometrial carcinomas and endometriosis all result from endometrial over-proliferation. Indeed, stem cell transformations may be the underlying cause leading to ovarian cancer. Based on these findings, we can conclude that bone marrow-derived stem cells could also contribute to endometriosis and may be relevant to the formation of endometrial carcinomas.

## Future directions

### Stem cells combined with biomaterials

In search of ways to achieve the sustained release of stem cell derivatives, as well as the long-term retention of stem cells in the body, more and more researchers have gravitated to the field of biomaterials. Specifically, researchers are combining biocompatible and biodegradable materials with stem cells or stem cell by-products to try to achieve better treatments. Ayala *et al*. [42] have engineered a patch made from multi-layered collagen sheets, impregnated with alginate gel, containing human MSCs. These patches are being used in studies that aim to restore abdominal wall defects without creating peritoneal adhesions in rats. Cao *et al*. [17] conducted a phase I clinical trial with 26 patients and a 30-month follow-up. In this trial, a collagen scaffold, loaded with umbilical cord-derived mesenchymal stromal cells (UC-MSCs), was used to prevent recurrent IUA after separation surgery. Three months after the stem cells were injected, as a result of successful endometrial and angiogenic proliferation, 10 of the 26 patients became pregnant and 8 of the 10 patients delivered successfully, without placental complications. The material used in this study was a degradable collagen scaffold that was transplanted into the uterine cavity after adhesiolysis surgery. Once the scaffold was in place, hormone replacement therapy was applied to induce and promote a natural menstrual cycle in all the patients. The umbilical cord-derived cells in this research were obtained noninvasively. The collagen scaffold served as an effective carrier, holding the UC-MSCs in place during the therapy. However, this clinical trial had no control group, so it is not known if the separation surgery alone would have had similar results, or if the UC-MSC-loaded collagen scaffold and/or the hormone replacement treatment made a difference.

### Synthetic stem cells

Stem cells applied clinically need to be carefully preserved before they are used. Additionally, transplanting stem cells directly into human beings carries immunogenic and tumorigenic risks. Based on the prevailing theory that stem cells exert their therapeutic benefits mainly through the secretion of growth factors and other protein/molecules, our group has fabricated a synthetic cell-mimicking microparticle (CMMP) to treat myocardial infarction [43]. The CMMP contains stem cell factors which have been surrounded by a stem cell membrane. This structure not only releases factors that support tissue regeneration but also avoids stimulating the immune reaction of T-cells. Although this study targets the heart, this CMMP strategy can be applied to various organs or tissues, including the uterus, which would broaden the role of stem cell-based therapies for the treatment of AS.

### Stem cell-derived factors

There are numerous growth factors related to the proliferation of epithelial and stromal endometrial cells. Transforming growth factor-alpha (TGFα), epithelial growth factor and platelet-derived growth factor-beta, beta, for example, strongly support epithelial and stromal cell growth. bFGF also promotes the growth of stromal cells. MSCs can secrete many factors that are related to endometrial proliferation. For example, hepatocyte growth factor (HGF), a MSC-derived growth factor, can contribute to the formation of the lumen in human endometrial epithelial cells. In addition, HGF has been shown to be an important mediator in the reconstruction of endometrial glandular elements [44]. Other MSC factors, like insulin-like growth factor 1 and TGFα are also associated with the proliferation of uterine epithelial cells.

### Stem cell-derived extracellular vesicles

Cellular secretions were credited with being responsible for the health benefits attributed to cellular therapies. Expanding on that premise, scientists are working to develop a therapy for AS that utilizes only the secreted extracellular vesicles (EV) of cells; among them exosomes. Although the current literature base for this topic is small, one group is using harvested EV from umbilical cord-derived MSCs to treat AS in combination with estrogen treatment [45]. The group isolates the exosomes from the other EVs present in the cell conditioned media using ultracentrifugation. Once isolated, they were administered into Albino rats with endometrial injury. In this study, the rats that received both exosome and estrogen applications experienced endometrial regeneration and a decrease in fibrotic manifestation. Despite this success, however, a number of questions remained unanswered. One question, which was also addressed in the paper itself, is whether or not the treatment would lead to higher fertility rates in the future. This gap was left there to be filled by future research endeavors. On a more mechanistic front, it would be of great value to understand what components within the exosomes, be they proteins or nucleic acids (i.e. miRNAs), are the greatest contributors to the regenerative process.

## Conclusions

**Figure 2.**
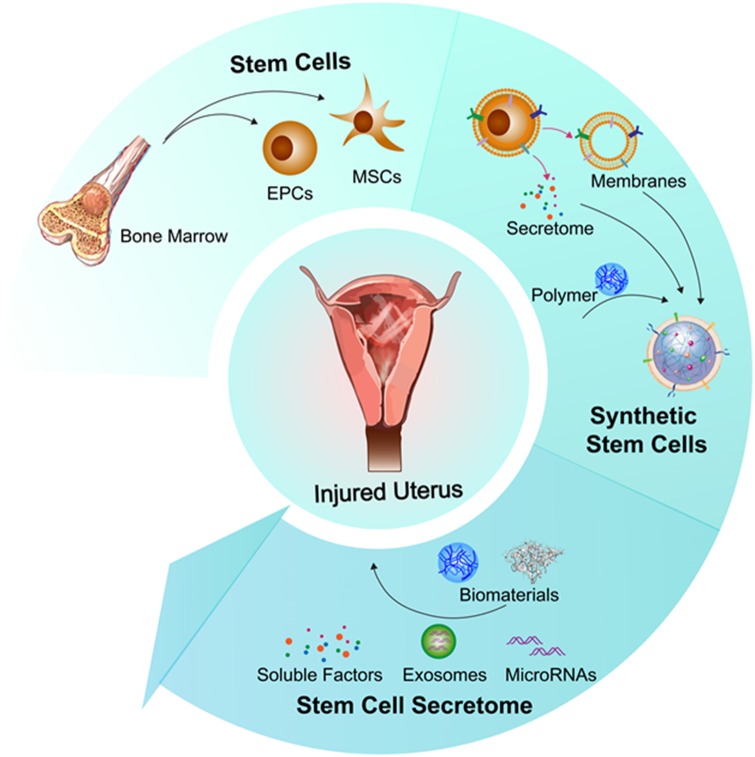
Regenerative medicine and biomaterials approaches to treat injured uterus, including (i) direct injection of bone marrow-derived stem cells; (ii) fabrication of synthetic stem cells by encapsulating stem cell therapeutics with biomaterials; (iii) engineering of nano-sized therapeutics by integrating biomaterials with stem cell secretomes. EPC, endothelial progenitor cell. MSC, mesenchymal stem cell.

AS is not an uncommon postoperative complication of intrauterine surgery. The etiology of this disease includes hypoxic injury, inflammatory response and deficient angiogenesis. A new theory links a deficient endometrial cell population with the onset of AS. Different treatments seek to reverse the mechanisms involved in IUAs. Hysteroscopy, hormone therapy and the use of intrauterine devices are some of the traditional methods which have been in use for some decades. Because AS takes place after endometrial injury, we can also use regenerative medicine to restore the injured endometrium and prevent the formation of AS. Biomaterials and stem cell therapies are significant components of regenerative medicine and play increasingly important roles in the treatment of AS. A growing number of animal experiments and clinical trials are focusing on the use of biomaterials to load stem cells as a treatment for AS. The use of encapsulated stem cell secreted growth factors, combined with biomaterials, is a very promising strategy to treat AS and prevent immune reactions, but more research and development is needed. What we should not ignore, however, are the risks of stem cell therapy. These include the possibility of immunogenicity and tumorigenicity. A breakdown of stem cell therapy options for AS is presented in the summary figure (Fig. 2).
